# Extracellular vesicles in normal pregnancy and pregnancy‐related diseases

**DOI:** 10.1111/jcmm.15144

**Published:** 2020-03-16

**Authors:** Jiayin Zhang, Haibo Li, Boyue Fan, Wenrong Xu, Xu Zhang

**Affiliations:** ^1^ Jiangsu Key Laboratory of Medical Science and Laboratory Medicine School of Medicine Jiangsu University Zhenjiang China; ^2^ Department of Clinical Laboratory Nantong Maternal and Child Health Care Hospital Nantong China

**Keywords:** biomarkers, complications, exosomes, extracellular vesicles, pregnancy

## Abstract

Extracellular vesicles (EVs) are nanosized, membranous vesicles released by almost all types of cells. Extracellular vesicles can be classified into distinct subtypes according to their sizes, origins and functions. Extracellular vesicles play important roles in intercellular communication through the transfer of a wide spectrum of bioactive molecules, contributing to the regulation of diverse physiological and pathological processes. Recently, it has been established that EVs mediate foetal‐maternal communication across gestation. Abnormal changes in EVs have been reported to be critically involved in pregnancy‐related diseases. Moreover, EVs have shown great potential to serve as biomarkers for the diagnosis of pregnancy‐related diseases. In this review, we discussed about the roles of EVs in normal pregnancy and how changes in EVs led to complicated pregnancy with an emphasis on their values in predicting and monitoring of pregnancy‐related diseases.

## 1 INTRODUCTION

During the process of pregnancy, a large range of extracellular vesicles (EVs) with different sizes are extruded into the maternal circulation, including macro‐vesicles (also termed as syncytial nuclear aggregates or mononuclear trophoblasts), micro‐vesicles (MVs) and exosomes.^1^, ^2^, ^3^ These distinct EVs could be distinguished by their specific features, including size, biogenesis process and biological functions.^4^, ^5^ Exosomes, with 30‐150 nm in diameter and from endocytic origin, are the most studied EVs so far. Extracellular vesicles contribute to cell‐to‐cell communication by transporting signalling molecules including proteins and nucleic acids. The cargos in these small vesicles may reflect the physiological or pathophysiological state of the source cells.^6^, ^7^ Emerging evidence suggests that exosomes with functional cargos can be transferred between foetus and maternal bodies.^8^ A variety of body fluids such as blood, urine and amniotic fluid contain EVs released from trophoblast cells, immune cells and endothelial cells, among others.^9^, ^10^, ^11^ EVs are suggested as essential modulators of multiple processes of pregnancy, including implantation, migration and invasion of trophoblasts, and cellular adaptations to the physiological changes.^1^, ^2^, ^3^ Changes in the concentration, composition and bioactivity of EVs have been reported to be associated with pregnancy‐related diseases.^12^, ^13^ Given these important roles, EVs have great potential to be developed as non‐invasive biomarkers of foetal and maternal conditions. In this review, we summarized the current knowledge about the functions of EVs in pregnancy and its complications. The contribution of macro‐vesicles to normal and complicated pregnancies has been well documented in other literatures.^14^, ^15^ Therefore, we mainly focused on the roles of exosomes and MVs in normal pregnancy and its related diseases. The clinical values of EVs detection for the early diagnosis and monitoring of pregnancy‐related diseases are also discussed.

## 2 EVs IN NORMAL PREGNANCY

**Figure 1:**
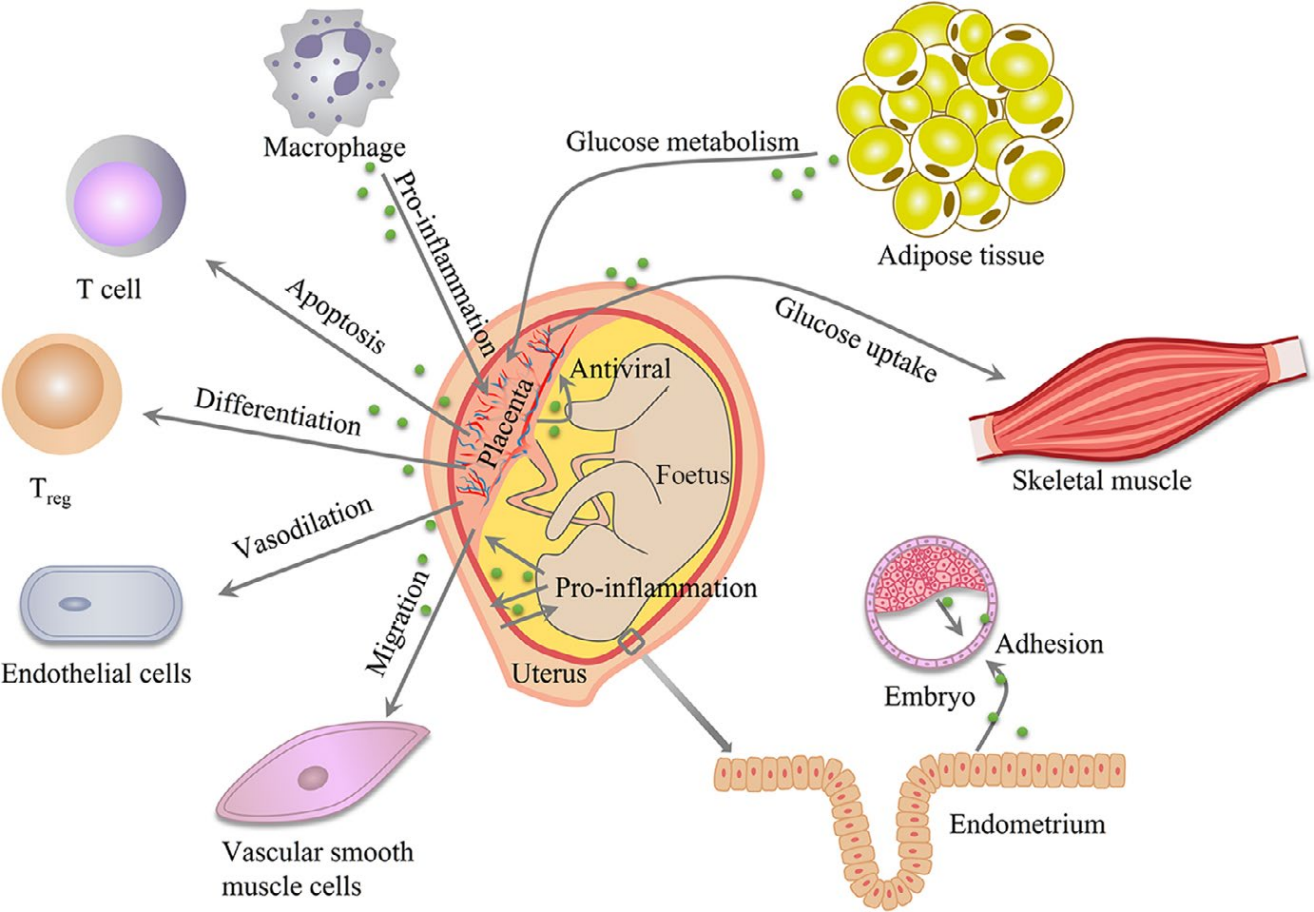
Effects of EVs in normal pregnancy. EVs mediate foetal‐maternal communications in normal pregnancy. EVs contribute to embryo implantation by promoting trophoblast adhesion. Placenta can interact with immune cells via EVs to balance immune activation and suppression across the gestation. EVs can activate endothelial cell (ECs) and vascular smooth muscle cells (VSMCs) to promote angiogenesis. EVs can accelerate glucose metabolism in the placenta and skeletal muscle. Moreover, inflammation signals of maturation in EVs can prepare uterus for delivery

Extracellular vesicles mediate foetal‐maternal communications and participate in many important physiological activities during normal pregnancy, including embryo implantation, immunomodulation, spiral arteries remodelling, metabolism adaptations and delivery (Figure 1).

### 2.1 EVs in embryo implantation

Extracellular vesicles take part in maternal‐embryo interaction within human uterine microenvironment, promoting implantation, an earliest and essential step for successful pregnancy.^16^, ^17^ Greening et al^18^ detected the proteome of exosomes from endometrial epithelial cells (ECs) that were treated with oestrogen (E; proliferative phase) or oestrogen plus progesterone (EP; receptive phase). The proteins enriched in exosomes of EP‐treated ECs are associated with embryo implantation and extracellular matrix remodelling, including fibulin1 (FBLN1), cysteine‐rich 61 (CYR61), laminin α5 (LAMA5) and collagen type XV (COL15A1). Exosomes from EP‐treated ECs could be taken up by trophoblast cells, promoting their adhesive capacity via activating focal adhesion kinase (FAK) signalling pathway. MicroRNAs (miRNAs) have been introduced as mediators of embryo‐endometrium crosstalk in the implantation process.^19^ MiRNAs packaged in MVs could be detected in human uterine luminal fluid, indicating their potential role in implantation.^20^, ^21^ Vilella et al^22^ provide evidence that miR‐30d is present in exosomes and could be transferred from receptive endometrial epithelium to embryo trophectoderm, improving the adhesive ability of pre‐implantation embryo. In addition, exosomal miR‐30d could act as a transcriptomic regulator, leading to overexpression of genes involved in embryo adhesion, such as Itgb3, Itga7 and Cdh5. The uptake of exosomal miR‐30d increases the rate of murine embryonic adhesion to the endometrial epithelium in vitro. They further demonstrate that maternal and/or embryonic miR‐30d deficiency impairs endometrial receptivity and embryo implantation rates in vivo by using wild‐type and miR‐30d knockout mice.^23^ Embryo itself can also generate MVs to enhance implantation by promoting the migration of trophoblast cells. Desrochers et al^24^ demonstrate that extracellular matrix proteins fibronectin and laminin α5 on the surface of MVs from embryonic stem cells can bind to integrin α5β1 and laminin receptors on trophoblast cells to trigger the activation of FAK and JNK signalling pathways. Moreover, they confirm that the implantation efficiency is increased in surrogate mice after injecting embryonic stem cell‐derived MVs into blastocysts, indicating an important role of EVs in mediating embryo implantation.

### 2.2 EVs in immunomodulation

Recent studies have highlighted a critical interaction between EVs and immune cells in modulation of pregnancy during which maternal immune system tolerates the growing foetus and maintains its normal functions.^25^, ^26^ Tong et al^27^ performed proteomic analyses of macro‐, micro‐ and nano‐extracellular vesicles derived from 56 first trimester placenta by using an ex vivo placental explant culture model. Gene Ontology pathway analysis shows an enrichment of proteins involved in vesicle transport and inflammation in all three fractions of EVs, which supports the notion that EVs can influence immune system during early pregnancy. Stenqvist et al^28^ demonstrate that placental exosomes carry active TNF superfamily members, including Fas ligand (FasL) and TNF‐related apoptosis‐inducing ligand (TRAIL), which plays an immune‐suppressive role through triggering apoptosis in activated peripheral blood mononuclear cells (PBMCs). The exposure to villous cytotrophoblast (VCT) exosomes suppresses PBMC activation. However, this effect is not observed in syncytin‐2 (Syn‐2)‐silenced VCT exosomes, indicating an important function of exosomal Syn‐2 in immune suppression.^29^ Kovacs et al^30^ suggest that trophoblastic EVs act as important players in immune tolerance, which is associated with their regulation of T_reg_ differentiation. Extracellular vesicle‐derived heat shock protein family E member 1 (HSPE1) promotes T_reg_ differentiation from CD4^+^ T cells and T_reg_ cell expansion in vitro*.* Placental exosome‐derived miR‐499 is elevated in the first trimester in cows and could inhibit NF‐κB activation via Lin28B/let‐7 axis, thereby repressing inflammation response and forming an immune‐tolerant microenvironment in the uterus. miR‐499 inhibition leads to inflammation dysregulation and increased risk of pregnancy failure in vivo*.* 31 Maternal immune system is essential for the uterus to prevent pathogenic infections.^32^ Holder et al^33^ demonstrate that macrophage derived exosomes can be internalized by placental cells via clathrin‐dependent endocytosis, increasing the release of pro‐inflammatory cytokines such as IL‐6, IL‐8 and IL‐10, potentially facilitating protective placental immune responses during pregnancy. Moreover, Delorme‐Axford et al^34^ demonstrate that primary human placental trophoblasts (PHTs) have antiviral immunity and can confer this resistance to non‐placental cells via PHT‐derived exosomes directly, shielding against viral infection during pregnancy. Primary human placental trophoblast‐derived exosomes package the chromosome 19 miRNA cluster (C19MC) miRNAs, which are highly expressed and specific in human placenta, to attenuate viral replication in recipient non‐placental cells by up‐regulating autophagy.

### 2.3 EVs in spiral artery remodelling and vascular function

To meet increased metabolic demands of the mother and foetus and to ensure adequate nutrients and oxygen supplies to the growing foetus, early stage of pregnancy requires sufficient spiral artery remodelling and physiological adaptations in the cardiovascular system.^35^, ^36^ Vascular smooth muscle cell (VSMC) migration is an important process during human uterine spiral artery (SpA) remodelling, which contributes to the successful pregnancy. Salomon et al^37^ show that exosomes isolated from extravillous trophoblast (EVT) cell lines (JEG‐3 and HTR‐8/SVneo cells) are capable of promoting VSMC migration, suggesting that EVT participate in SpA remodelling through exosomes‐mediated promotion of VSMC migration out of the vessel walls. They further show that endothelial cell migration in vitro can be stimulated by circulating exosomes from pregnant women. Intriguingly, the bioactivity of exosomes is greatest during first trimester and gradually decline with increasing gestational age.^38^ Tong et al^39^ demonstrate that different size fractions of placental EVs can rapidly interact with ECs and localize to different organs in vivo, which supports the notion that placental EVs are likely to have effects on endothelial functions. Placental nano‐vesicles interact with ECs rapidly in vitro through a combination of mechanisms including phagocytosis, endocytosis and cell surface binding. Interestingly, the nano‐vesicles extruded from first trimester human placenta cannot affect endothelium‐dependent vasodilation of uterine artery whereas it affects the ability of systemic mesenteric arteries to undergo endothelium‐ and nitric oxide‐dependent vasodilation in pregnant mice.^40^ There is evidence that normal circulating piglet foetal exosomes derived from the umbilical vein are able to increase tube formation function of human umbilical vein endothelial cells (HUVECs). After coculture with normal circulating piglet foetal exosomes derived from the umbilical vein, the expression of pro‐angiogenic genes VEGF and Notch1 are up‐regulated while that of anti‐angiogenic gene TSP1 is down‐regulated in HUVECs.^41^ Similarly, Jia et al^42^ provide evidence that both maternal and umbilical serum exosomes enhance HUVEC proliferation, migration and tube formation abilities. Furthermore, the altered expression of a subset of migration‐related miRNAs, including miR‐210‐3p, miR‐376c‐3p, miR‐151a‐5p, miR‐296‐5p, miR‐122‐5p and miR‐550a‐5p, has been identified in umbilical serum exosomes.

### 2.4 EVs in metabolism

Increasing evidence suggests that EVs are involved in metabolic homoeostasis.^43^, ^44^ However, little is known about the role of EVs in metabolic regulation with regard to pregnancy. Nair et al^45^ demonstrate that placental exosomes from normal glucose tolerant (NGT) pregnant women are able to increase insulin‐induced glucose uptake in skeletal muscle from diabetic patients, suggesting placental exosomes may engage in the changes of insulin sensitivity in normal pregnancies. Recently, Jeyabalan and colleagues demonstrate that treatment with exosomes from adipose tissue (AT) of NGT pregnant women affects the expression of glucose metabolism‐related genes in placental cells. They provide data that the up‐regulated genes in placental cells are associated with glycolysis (HK3 and TPI1), gluconeogenesis (PCK1 and G6PC), glycogen production (UGP2) and degradation (PYGM), suggesting that physiological adaptation by exosomes could satisfy increasing glucose usage in foetus and placenta across pregnancy.^46^

### 2.5 EVs in delivery

Signals of foetus maturation probably induce inflammatory responses and thus prepare the uterus for delivery.^47^, ^48^ The previous study has shown that the activated form of p38 mitogen‐activated protein kinase (MAPK) is a term parturition associated marker.^49^, ^50^ Sheller et al^51^ demonstrate that phosphorylated p38 MAPK is expressed in exosomes from amnion epithelial cells (AECs) and is up‐regulated in response to oxidative stress. Hadley et al^52^ have tested if senescent foetal AEC exosomes can cause inflammatory changes in maternal and placental tissues. Primary AECs are grown in normal cell culture (control) or oxidative stress condition, and myometrial and decidual cells are treated with those AEC‐derived exosomes. Regardless of the source of exosomes, exosome treatments increase the secretion of IL‐6, IL‐8 and prostaglandin E2 (PGE2) and induce the activation of NF‐κB in uterine myometrial and decidual cells. However, exosomes produced under oxidative stress condition have a more dominant pro‐inflammatory effect than control exosomes. In addition, more foetal exosomes are possible to reach maternal gestational tissues at term labour. Altogether, their data imply that foetal cell exosomes may contribute to the timing of birth by increasing uterine cell inflammation. Ithier et al^53^, ^54^ suggest that foetal lung‐derived C4BPA plays a role in birth timing determination by utilizing proteomics analysis of exosomes from foetal cord arterial blood. They reveal that C4BPA could bind to CD40 of placental villous trophoblast to promote p100 processing to p52. In turn, RelB/p52 heterodimers transport to the nucleus to activate non‐canonical NF‐κB pathway in placenta, which drives the epigenetic changes in pro‐labour genes. A recent study in mouse model also suggests that non‐specific inflammation builds up gradually across late gestation and reaches the peak at the day before the expected delivery. Exosomes from uterine tissues at late gestation contain pro‐inflammatory cargos and could affect birth timing by promoting local inflammatory responses in foetal membranes.^55^

## 3 EVs IN COMPLICATED PREGNANCY

**Figure 2:**
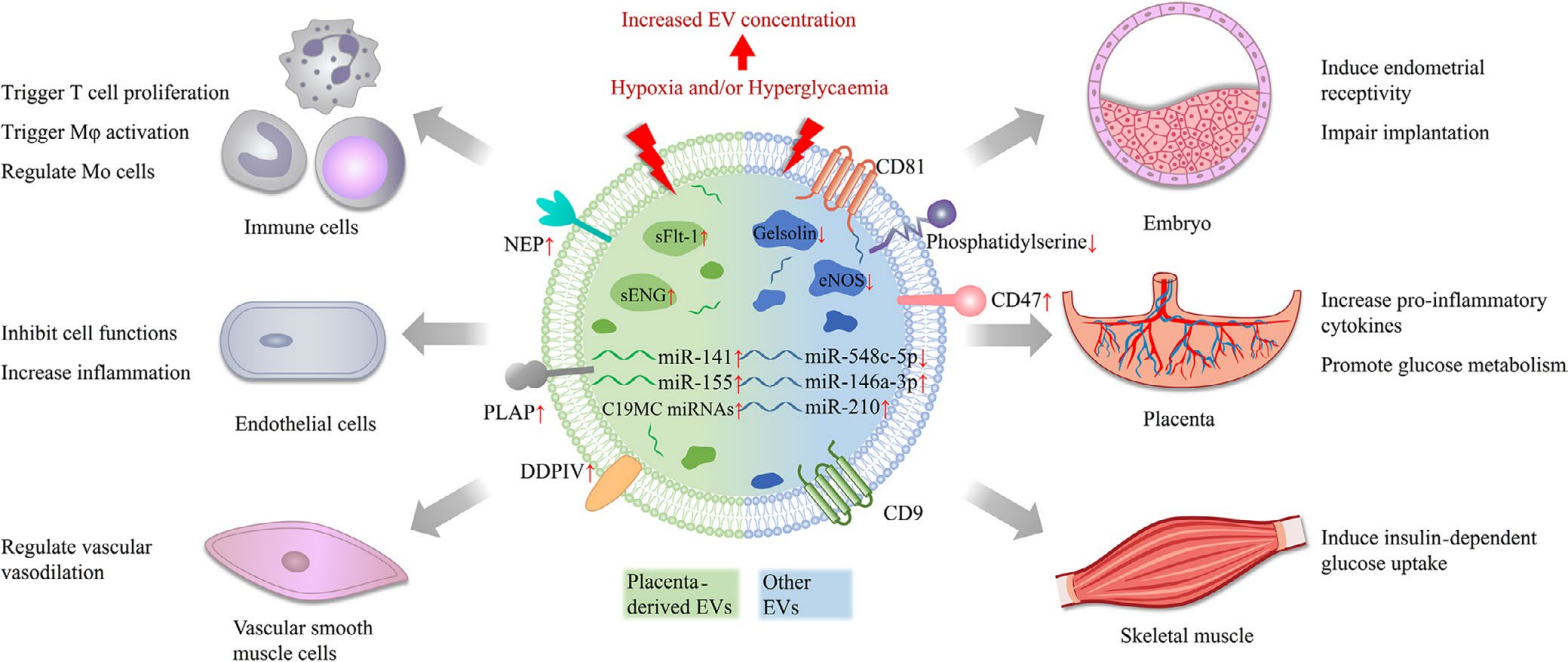
Effects of EVs in pregnancy‐related diseases. Abnormal changes of EVs concentration and composition are involved in pregnancy‐related diseases. EVs mediate dysregulation of the balance between pro‐ and anti‐inflammatory responses in immune cells, ECs and placenta. EVs can lead to failures in endothelial functions and vasoconstriction. Communications between placenta and important metabolism tissues via EVs are correlated with glucose metabolism and insulin resistance. Moreover, EVs can decrease implantation efficiency by inducing endometrial receptivity

Abnormal changes of EV concentration and composition are involved in pregnancy‐related diseases, including pre‐eclampsia (PE), gestational diabetes mellitus (GDM), preterm birth (PTB) and other adverse pregnancy outcome (Figure 2).

### 3.1 EVs in inflammation

The dysregulation of the balance between pro‐ and anti‐inflammatory factors has been reported in complications of pregnancy.^56^, ^57^ Exosomes from extravillous trophoblast cells cultured under low oxygen tension could increase TNFα expression in HUVECs, thus inhibiting their migration.^58^ In addition, EV‐induced active platelets could activate NLRP3 inflammasome in trophoblast cells via ATP‐purinergic signalling, leading to PE‐like symptoms (ie pregnancy failure, elevated blood pressure, increased plasma sflt‐1 and renal dysfunction) in mice. Notably, platelets induce placental sterile inflammation without blood clots and increase fibrin accumulation, indicating that EVs‐activated platelets are linked with inflammatory reaction directly in this setting.^59^ In PE patients, the level of plasma gelsolin, an anti‐inflammatory factor of maternal origin, is much lower than that in healthy women at late stage of pregnancy, which may be associated with the shedding of EVs.^60^ The expression of high mobility group box 1 (HMGB1), a pro‐inflammatory danger signal, is increased in macro‐vesicles derived from trophoblast after treating placental explants with pre‐eclamptic sera.^61^ Ospina‐Prieto et al^62^, ^63^ show that exosomal miR‐141 derived from foetal trophoblast is elevated in PE patients and miR‐141‐enriched trophoblast exosomes could induce T cell proliferation, indicating that placental EVs regulate maternal immune cells and cause immune disorders in pregnancy as PE is associated with systemic pro‐inflammatory environment. Kovacs et al^64^ demonstrate that PE‐MVs could interact with monocytes and modify their phenotype and function. An altered phagocytosis‐associated molecular pattern is found in PE‐EVs, including an elevated CD47 ‘don't eat me’ signal and a decreased exofacial phosphatidylserine ‘eat me’ signal along with decreased uptake of PE‐EVs. Compared to healthy pregnancy MVs, PE‐MVs suppress the chemotactic activity and the motility of monocytes and accelerate their adhesion. MiR‐548c‐5p is lowly expressed in serum exosomes and placental mononuclear cells in PE patients compared to normal pregnancies. MiR‐548c‐5p down‐regulation is associated with increased secretion of inflammatory cytokines (IL‐12 and TNF‐α) and nuclear translocation of NF‐κB in macrophages, which in turn triggers the proliferation and activation of macrophages and stimulates inflammatory response.^65^ Exosomes from GDM pregnancies increase the release of pro‐inflammatory cytokines from ECs, including GM‐CSF, IL‐6 and IL‐8, among others,^66^ similar to exosomes isolated from cells cultured under high glucose condition.^67^ The levels of pro‐inflammatory cytokines are positively associated with maternal BMI.^68^ Moreover, the inflammatory cargos in exosomes are involved in preterm labour (PTB) by inducing a localized inflammatory response in mice.^55^ By using sequential windowed acquisition of all theoretical mass spectra (SWATH‐MS) to screen differentially expressed proteins in circulating maternal exosomes, Menon et al^69^ show that inflammation‐associated molecules are enriched in PTB exosomes, suggesting that inflammation likely affects labour process through exosomes. Gysler et al^70^ reveal that the level of plasma exosomal miR‐146a‐3p is higher in pregnant women with antiphospholipid antibody (aPL) and adverse pregnancy outcome (APO) than those with aPL but without APO at 18‐27 weeks of gestation. They suggest that miR‐146a‐3p is a mediator of aPL‐induced IL‐8 secretion in trophoblast cells, thus exerting a pro‐inflammatory effect on the placenta.

### 3.2 EVs in vascular dysfunction

Failures in endothelial functions and vasoconstriction responses increase the risk of vascular complications of pregnancy, especially PE.^71^, ^72^ Exosomes from PE patients contain abundant placental anti‐angiogenic factors such as soluble fms‐like tyrosine kinase‐1 (sFlt‐1) and soluble endoglin (sEng). These exosomes could be internalized by HUVECs and impair their proliferation, migration and tube formation functions in vitro. Similarly, these exosomes cause vascular dysfunction and result in adverse pre‐eclampsia‐like birth outcome in mice.^73^ Cronqvist et al^74^ provide evidence that placenta‐specific C19MC miRNAs derived from syncytiotrophoblast‐derived extracellular vesicles (STBEVs) could be transferred into the endoplasmic reticulum (ER) and mitochondria of primary human ECs. When ECs are treated with PE derived STBEVs, the miRNA deposition is altered from the mitochondria to the ER and the cell membrane becomes ruffled. More importantly, the miRNAs are functional, causing a down‐regulation of specific target genes including the PE‐associated gene Flt‐1. Interestingly, Ermini et al^75^ suggest that circulating form of Eng is not a soluble protein but a truncated extracellular domain of Eng. Circulating Eng are secreted from the syncytiotrophoblast (STB) via exosomes together with types 1 and 2 TGF‐β receptors. This complex affects vascular homeostasis and induces hypertension through impairing TGF‐β signalling. In PE, syncytiotrophoblast may extrude dangerous macro‐vesicles that can subsequently trigger EC dysfunction. For instance, aPL, the strongest maternal risk factor for PE, can result in an increased extrusion of macro‐vesicles derived from trophoblast. In addition, the expression of 72 proteins in those macro‐vesicles is altered, of which 13 proteins are involved in mitochondrial function. These altered macro‐vesicles may trigger endothelial dysfunction and PE in pregnancies.^76^, ^77^ Foetal growth restriction (FGR) may occur due to repressed angiogenesis and insufficient blood supplement. When HUVECs are treated with circulating piglet foetal exosomes derived from the umbilical vein under FGR condition, the expression of pro‐angiogenic genes VEGF and Notch1 is down‐regulated while that of anti‐angiogenic gene TSP1 is up‐regulated. Furthermore, this study reveals that a lower level of exosomal miR‐150 is associated with decreased proliferation, migration and tube formation of HUVECs.^41^ A number of studies suggest that placental STBEVs exert influence on maternal vasoconstriction responses across gestation. Spaans et al^78^ suggest that STBEVs impair angiotensin II^79^ and NO^78^‐mediated vasodilation through lectin‐like oxidized low‐density lipoprotein receptor‐1 (LOX‐1) in ECs. They suggest that the inhibition of LOX‐1 increases endothelial nitric oxide synthase (eNOS) expression in STBEV‐incubated vessels. The decreased eNOS level is considered to be associated with decreased bioavailability of NO in PE. Motta‐Mejia et al^80^ demonstrate that STBEVs isolated from both placenta perfusate and circulating plasma of PE have lower levels of STBEV‐eNOS compared to normal pregnant group. Placental exosomes from PE patients reduce eNOS expression and NO production in HUVECs, which could be attributed to increased expression of miR‐155 in exosomes.^81^ Neprilysin (NEP) is a membrane‐bound metalloprotease that could reduce the activation of peptides such as vasodilators, natriuretics and diuretics. A recent study suggests that active NEP is highly expressed in STBEVs of PE patients, which are likely to be associated with the pathogenesis of PE.^82^

### 3.3 EVs in metabolic disorders

Metabolism dysregulation is closely related to some pregnant complications, for instance, GDM and foetal overgrowth.^83^, ^84^ Compared to AT exosomes of NGT controls, AT exosomes of GDM could increase the expression of genes associated with glycolysis and glycogenolysis in placental cells. It is possible that increased placental glycogenolysis in GDM accelerates glucose transferring to foetus, resulting in foetal overgrowth. Furthermore, ingenuity pathway analysis reveals the differential expression of mitochondrial function‐related proteins in AT exosomes of GDM.^46^ The work from Nair et al^45^ shows that placental exosomes are capable of modulating insulin‐stimulated migration and glucose uptake in primary skeletal muscle cells. Gene target and ontology analyses of differentially expressed miRNAs in GDM exosomes show that they are associated with pathways regulating cell migration and carbohydrate metabolism. These findings imply that EV‐mediated communications between placenta and important metabolism tissues (adipose tissue and skeletal muscle) are closely associated with glucose metabolism and insulin resistance during pregnancy.

## 4 EVs AS BIOMARKERS FOR PREGNANCY‐RELATED DISEASES

Extracellular vesicles have a tissue‐specific pattern and may reflect the status of source cells, which highlights their usefulness as indicators of cellular function and as biomarkers for various diseases. Increasing studies suggest that EV concentration and content are of great diagnostic utility for women at risk of pregnancy‐related diseases (Table 1).

**Table 1:** The clinical values of EVs in pregnancy complications

Pregnancy complications	Source of EVs	Pregnancy stage	Targets	Isolation method	Detection method	Clinical value	References
Pre‐eclampsia	Plasma	Early	Total exosomes, exosomal PLAP	Ultracentrifugation, differential centrifugation	ELISA	Elevated in PE at early pregnancy (AUC 0.745 and 0.829)	85
	Plasma	Late	Exosomal PLAP to total exosomes ratio	Ultracentrifugation	ELISA	Reduced in PE; lower in late‐onset PE than early‐onset PE	86
	Plasma	Late	miRNAs profile	Commercial kit	Nanostring counter system miRNA assay	Potential markers of PE and subtypes of PE	88
	Plasma	Mid and late	miR‐210	Commercial kit	qRT‐PCR	Elevated in PE; higher in severe PE	89
	Plasma	Early	miR‐486‐1‐5p, miR‐486‐2‐5p	Ultracentrifugation, differential centrifugation	RNA sequencing	Elevated in PE at early pregnancy	85
	Plasma	Mid and late	miR‐136, miR‐494, miR‐495	Ultracentrifugation	qRT‐PCR	6.4‐, 3.9‐ and 2.1‐fold higher in PE than normal pregnancy	91
	Serum	Late	miR‐155	Ultracentrifugation, differential centrifugation	qRT‐PCR	Elevated in PE	81
	Serum	Late	miR‐548c‐5p	Commercial kit	qRT‐PCR	Reduced in PE	65
	Plasma	Early	miR‐517‐5p, miR‐520a‐5p, miR‐525‐5p	Commercial kit	qRT‐PCR	Reduced in PE at early pregnancy (AUC 0.719)	92
	Plasma	Late	PLAP^+^NEP^+^ EVs	Size exclusion chromatography	FCM	Elevated in PE	82
	Urine	Late	Podocin^+^ EVs‐to‐nephrin^+^ EVs ratio	Without isolation	FCM	Elevated in PE; correlated with renal injury	94
	Urine	Late	ENaC, NKCC2	Differential centrifugation	WB	Elevated in PE; correlated with renal injury	96
Gestational diabetes mellitus	Plasma	Early, mid and late	PLAP^+^EVs	Ultracentrifugation, differential centrifugation	ELISA	2.2‐fold higher at early gestation in GDM than normal pregnancy	66
	Plasma	Early, mid and late	PLAP per exosome	Ultracentrifugation, differential centrifugation	ELISA	63% lower at early gestation in GDM than normal pregnancy	66
	Oral fluid	Early	Total exosomes	Commercial kit	NTA	Elevated in GDM at early pregnancy (AUC 0.81)	97
	Plasma	Late	miR‐125a‐3p, miR‐99b‐5p, miR‐197‐3p, miR‐22‐3p, miR‐224‐5p	Ultracentrifugation, differential centrifugation	RNA sequencing, qRT‐PCR	Elevated in GDM; related to metabolism	45
	Serum	Early	10 miRNAs	Differential centrifugation	qRT‐PCR	Elevated in GDM at early pregnancy	98
	Plasma	Late	DPPIV^+^PLAP^+^ EVs	Without isolation	FCM	Eightfold higher in GDM than normal pregnancy	99
	Plasma	Late	78 proteins	Ultracentrifugation	SWATH‐MS	Potential markers of GDM	100
Preterm birth	Plasma	Early, mid and late	173 miRNAs	Ultracentrifugation	RNA sequencing	Potential markers of PTB	103
	Urine	Late	16S rRNAs derived from Ureaplasma and Veillonellaceae	Differential centrifugation	RNA sequencing	Elevated in PTB	104
	Plasma	Late	72 proteins	Differential centrifugation, size exclusion chromatography	SWATH‐MS	Potential markers of PTB	69
	Plasma	Early	62 proteins	Size exclusion chromatography	LC‐MS	PTB predictor at early pregnancy	105
Foetal growth restriction	Plasma	Late	PLAP^+^ exosomes to total exosomes ratio	Ultracentrifugation, differential centrifugation	NTA	Reduced in FGR; corrected with birth weight percentile	106
	Serum	Mid	miR‐20b‐5p, miR‐942‐5p, miR‐324‐3p, miR‐223‐5p, miR‐127‐3p	‐	RNA sequencing	Elevated in FGR	107

Abbreviations: ELISA, enzyme‐linked immunosorbent assay; FCM, flow cytometry; LC‐MS, liquid chromatograph‐mass spectrometer; NTA, nanoparticle tracking analysis; qRT‐PCR, quantitative polymerase chain reaction; SWATH‐MS, sequential windowed acquisition of all theoretical mass spectra; WB, Western blotting.

### 4.1 Pre‐eclampsia

Salomon et al^85^ show that the concentrations of total exosome and exosomal placental alkaline phosphatase (PLAP), a placental marker, are up‐regulated in maternal plasma of PE at early, mid‐ and late gestation. Receiver operating characteristic curve (ROC) analysis is performed to assess the diagnostic potential of total exosome and exosomal PLAP concentrations at early pregnancy (11‐14 weeks). The areas under the ROC curve (AUC) for total exosome and exosomal PLAP concentrations are 0.745 and 0.829, respectively. Additionally, the relative concentration of placenta‐derived exosomes (ratio of exosomal PLAP to total exosome number) is different between early‐onset PE and late‐onset PE.^86^ A pioneer study demonstrates that EVs derived from injured placenta induce PE‐like symptoms like hypertension and proteinuria in mice by inducing endothelial injury, vasoconstriction and hypercoagulation. These symptoms are reversed by enhancing EV clearance, indicating therapeutic value of placental EV production and clearance rates.^87^ The analyses of exosomal miRNA profile in early‐ and late‐onset PE women plasma have identified several dysregulated miRNAs in PE and its subtypes. Therefore, EVs profile may have potential values to be used as diagnostic markers for PE and its subtypes.^88^ Biro et al^89^, ^90^ have detected serum exosomes from women with chronic hypertension (CHT), gestational hypertension (GHT), moderate PE, severe PE and normotensive pregnancies. The expression levels of miR‐210 are significantly increased in PE than that in other groups and are highest in the severe form of PE, showing miR‐210 may have an important role in the pathogenesis of PE. Twelve differentially expressed miRNAs are identified in PE pregnancies, among which miR‐486‐1‐5p and miR‐486‐2‐5p are suggested as candidate indicators of early stage PE.^85^ In peripheral blood from patients with PE, exosomal miR‐136, miR‐494 and miR‐495 are 6.4‐, 3.9‐ and 2.1‐fold higher than that in normal pregnancies, respectively, according to a study enrolling 100 PE patients.^91^ In addition, miR‐141,^62^ miR‐155^81^ and miR‐548c‐5p^65^ are detected with altered expression in PE and identified as the candidate miRNA markers. During the first trimester of pregnancy, miR‐517‐5p, miR‐520a‐5p and miR‐525‐5p are observed to be down‐regulated in plasma exosomes of PE patients with later occurrence. A panel of three placenta‐specific C19MC miRNAs shows modest diagnostic value with an AUC of 0.719.^92^ Neprilysin (NEP), a membrane‐bound metalloprotease, is highly expressed in STBEVs from the plasma of PE patients as detected by flow cytometry. Thus, the higher level of PLAP^+^NEP^+^ EVs is suggested as a promising biomarker of PE.^82^ Son et al^93^ demonstrate that the expression level of podocyte‐specific protein nephrin is elevated in the urine of PE women and is correlated with proteinuria and diastolic blood pressure. The expression level of nephrin is reduced in pre‐eclamptic renal tissue compared to that of normotensive pregnancies. The elevated urinary podocin^+^ EVs‐to‐nephrin^+^ EVs ratio is associated with renal injury in PE.^94^ In addition, abnormal increased sodium reabsorption also contributes to hypertension in PE. Impaired urine sodium excretion, Na/K ratio and elevated urine plasminogen are also found in PE.^95^ In urine EVs, increased expression of epithelial sodium channel (ENaC) and phosphorylation of Na‐Cl2‐K co‐transporter 2 (NKCC2) are observed in PE, indicating the status of renal dysfunction.^96^

### 4.2 Gestational diabetes mellitus

The aberrant changes in placental microenvironment may affect the release and content of placenta‐derived EVs. The quantity of exosomes secreted from trophoblast cells cultured under high glucose condition is approximately three times higher than that cultured under normal glucose condition.^67^ Salomon et al^66^ have evaluated the diagnostic potential of placental exosome concentration present in maternal plasma at early, mid‐ and late gestation. Compared to normal pregnancies, the concentration of placental exosome in GDM pregnancies increased about 2.2‐, 1.5‐ and 1.8‐fold at each point of gestation, which indicates that exosome profile is of diagnostic value to screen asymptomatic populations. PLAP content per exosome (PLAP ratio) is determined to estimate the relative contribution of placental exosomes to total exosomes. PLAP ratio is lower in GDM than normal pregnancy even though both placental exosomes and total exosomes are higher in GDM, which implies that there are changes in the number of exosomes released from the placenta, increased release of exosomes from non‐placental sources or a combination of both. Interestingly, Monteiro et al^97^ demonstrate that gingival crevicular fluid‐derived EVs can distinguish patients at risk of GDM. At 11‐14 weeks of gestation, the concentration of oral EVs is higher in asymptomatic women compared to controls with an AUC value of 0.81. Five metabolism‐related miRNAs, namely miR‐125a‐3p, miR‐99b‐5p, miR‐197‐3p, miR‐22‐3p and miR‐224‐5p, are consistently detected with high expression in skeletal muscle, placenta, placenta‐derived exosomes and circulating exosomes in GDM.^45^ Gillet et al^98^ have determined the miRNA profile of serum EVs in GDM at early pregnancies. Ten miRNAs (miR‐122‐5p; miR‐132‐3p; miR‐1323; miR‐136‐5p; miR‐182‐3p; miR‐210‐3p; miR‐29a‐3p; miR‐29b‐3p; miR‐342‐3p; and miR‐520h) show significantly higher levels in GDM cases compared to controls. Bioinformatics analysis indicates that these miRNAs are involved in trophoblast proliferation and differentiation, as well as insulin regulation and glucose transport in pregnant women. STBEVs carry active dipeptidyl peptidase IV (DPPIV), which is able to break down glucagon‐like peptide‐1 (GLP‐1) and then regulates glucose‐dependent insulin secretion. The concentration of DPPIV^+^ STBEVs in GDM maternal plasma is more than eightfold higher than that in normal pregnancies. Increased DPPIV biological activity is also demonstrated in STBEVs from GDM.^99^ A total of 78 exosomal proteins are differentially expressed in GDM compared to NGT. Bioinformatic analysis shows that the differentially expressed exosomal proteins in GDM are enriched in pathways associated with energy production, inflammation and metabolism.^100^

### 4.3 Preterm birth

Extracellular vesicles have been suggested as indicators of preterm birth (PTB), with an advantage of non‐invasive isolation from maternal blood and other biological fluids.^101^, ^102^ In a longitudinal study, Menon et al^103^ include a cohort of patients with term birth and PTB and reveal a total of 173 miRNAs with significant changes in circulating exosomes across three gestational period by using next‐generation sequencing. The altered miRNAs can be divided into several clusters with different trends of changes over time. As such, miRNA content of exosomes in maternal blood may represent as biomolecular ‘fingerprint’ of the pregnancy progression. Moreover, bacteria‐derived 16S rRNAs in urine EVs are capable of indicating pregnant status. Extracellular vesicles derived from *Ureaplasma* and *Veillonellaceae* are more abundant in the urine of PTB women than normal deliveries.^104^ Menon et al^69^ have compared the proteomes of maternal plasma exosomes among four groups, including term not in labour, term in labour, preterm premature rupture of membranes and PTB. They have identified 72 proteins with significant changes among these four groups. Similarly, Cantonwine et al^105^ have analysed circulating MVs from women between 10 and 12 weeks of gestation who subsequently develop spontaneous PTB at 34 weeks. Through ROC analysis with bootstrap resampling, they have identified 62 proteins qualified for diagnosis among 132 proteins evaluated. A panel of three exosomal proteins (A2MG, HEMO and MBL2) exhibits a specificity of 83% with a median AUC of 0.89. These candidates, if further validated, will allow the stratification of patients at risk of spontaneous PTB before clinical manifestation.

### 4.4 Foetal growth restriction

In a cohort study of pregnant women who give birth to small foetuses, Miranda et al^106^ have detected the concentrations of total and placental exosomes in the circulation by labelling CD63 and PLAP. The ratio of PLAP^+^CD63^+^ exosomes to PLAP^−^CD63^+^ exosomes is used to describe the contribution of placental exosomes to the total. The ratio is positively correlated with the percentile of birth weight and displays a clear trend according to the severity of the disease. Thus, relative concentration of placental exosomes may be used as a marker for foetal growth. Rodosthenous et al^107^ demonstrate that the expression levels of miR‐20b‐5p, miR‐942‐5p, miR‐324‐3p, miR‐223‐5p and miR‐127‐3p are higher in pregnant women at second trimester who have a small gestational age (SGA) infants later. MiR‐127‐3p exhibits the most robust association with abnormal foetal growth, which has not been evaluated in relation to foetal growth by the previous studies.

## 5 CONCLUSIONS

The current studies provide us a comprehensive understanding of EVs in normal and complicated pregnancy and an opportunity to develop novel methods for early and efficient diagnosis of pregnancy‐related diseases. Extracellular vesicles play diverse roles across the gestation such as embryo implantation, immunomodulation, vascular remodelling and metabolism adaptation by mediating foetal‐maternal crosstalk. The available data partially unravel the roles and underlying mechanisms of EVs in the physiology or pathophysiology of pregnancy. Extracellular vesicle‐derived bioactive molecules may explain their roles in regulating cellular functions and contribute to pregnancy‐related diseases. Moreover, the concentration and content of circulating EVs show potential diagnostic value as they may reflect condition changes, metabolism status, foetal growth and foetal maturation. The development of precise diagnosis may allow implementation of appropriate intervention, which would be helpful for reducing the harm to both mothers and foetus. However, there are still several problems that need to be addressed before EVs reach the clinic in human reproduction field. First, there are still no recognized common methods for the discrimination and quantification of different sub‐populations of EVs. It is necessary to establish stable and reliable methods to achieve rapid separation and automated analysis of EVs. Second, due to the difficulty of imitating long‐term effects in vitro and the lack of suitable cell and animal models, controversial and inconsistent results still exist in different studies. Moreover, human tissues usually only can be obtained after delivery and cannot accurately reflect the dynamic changes of markers during the whole pregnancy process. Therefore, more efforts should be devoted to giving an insight into the functions of EV in pregnancy and to apply EVs to the diagnosis, monitoring and treatment of pregnancy‐related diseases.

## CONFLICT OF INTEREST

The authors confirm that there are no conflicts of interest.

## AUTHORS' CONTRIBUTIONS

JY Zhang and X Zhang contributed to the conception of the manuscript. JY Zhang, BY Fan and HB Li collected literature and draft the manuscript. WR Xu and X Zhang reviewed and made significant revisions to the manuscript. All authors read and approved the final manuscript.
